# Supplementary material for: WordCommentsAnalyzer: A windows software tool for qualitative research
Source: F1000Res. 2018 Sep 4;7:536. Originally published 2018 May 3. [Version 2] doi: 10.12688/f1000research.14819.2 (PMC6137411; doi:10.12688/f1000research.14819.2)
Supplement: Supplementary file 3 [file f1000research-7-17614-s0002.tgz › b2a79496-4ada-47c4-9682-a99ce867836d.zip!/Eight Iranian Health Journals/Salmand_Iranian Journal of Ageing _38.docx]

© Salmand_Iranian Journal of Ageing . This work is licensed under CC BY-NC 4.0 (https://creativecommons.org/licenses/by-nc/4.0/).

Volume & Issue: دوره 4 - شماره 1

Title: بهداشت روان سالمند در قرآن و حدیث

http://salmandj.uswr.ac.ir/article-1-335-fa.html

اهداف: هدف از پژوهش حاضر بررسی بهداشت روانی سالمندان در قرآن و حدیث بود. مواد و روش‌ها: در این پژوهش، مضامین مرتبط با بهداشت روانی سالمندان در قرآن و حدیث شناسایی و با ساختاری آموزشی تدوین گردید. در این راستا قرآن کریم و کتب حدیثی با استفاده از نرم افزار حدیثی نور که مشتمل بر 442 جلد کتاب حدیثی است با استفاده از روش تحلیل محتوا و کتابخانه ای مورد بررسی قرار گرفت. به خاطر دستیابی به نظریات بنیادین دینی مضامین از کتاب قرآن و کتب حدیث که از متون و منابع اصلی دینی است گزینش گرید. یافته‌ها: نتایج پژوهش شامل، تعریف سالمندی از نگاه اسلام، معرفی سالمند، جایگاه سالمند، حقوق سالمند، حق والدین سالمند بر فرزندان، حق سالمند بر حکومت، اخلاق، آداب سالمندی، رفتار با سالمند، رفتار با والدین سالمند، «بر» و احسان و شیوه های احسان و «بر» رفتار معروف و مناسب با والدین، گفتار کریمانه رفتار متواضعانه، صله رحم، نفقه، دعا و شکرگزاری است که در متن مقاله هر کدام به تفصیل بیان شده است. نتیجه‌گیری: بر اساس نتایج به دست آمده در قرآن و حدیث می توان نتیجه گرفت بهداشت روانی سالمندان از سه جنبه مورد توجه قرار گرفته است: 1. سالمند از نگاه عموم مردم؛ 2. سالمند از نگاه حکومت؛ 3. سالمند از نگاه نقش والدینی آنان.

Title: تأثیر تمرین استقامتی بر برخی پارامترهای کینماتیک راه رفتن سالمندان غیر فعال

http://salmandj.uswr.ac.ir/article-1-334-fa.html

اهداف: هدف این پژوهش، بررسی تاثیر اجرای یک دوره برنامه تمرین استقامتی بر الگوی راه رفتن سالمندان غیر فعال بود. مواد و روش‌ها: این مطالعه به روش نیمه تجربی انجام شد. 23 زن و مرد سالمند با میانگین و انحراف معیار سنی 6/9±70/50 سال (در دو گروه 14 نفر گروه تمرین و نه نفر گروه کنترل) در این تحقیق شرکت کردند. گروه تمرین در برنامه تمرینی به مدت هشت هفته و سه جلسه در هفته شرکت کردند در حالیکه گروه کنترل به زندگی عادی خود پرداختند. مقادیر اندازه گیری شده شامل طول گام، آهنگ گام برداری، سرعت، درصد استقرار در فاز استانس و تغییرات دامنه حرکتی مفاصل اندام تحتانی توسط نرم افزارهای اتوکد 14، یولید 10، ویندوز مدیا پلیر و سی جی آ در دو مرحله قبل و بعد از برنامه ورزشی در دو گروه تمرین و کنترل به دست آمد سپس توسط نرم افزاز SPSS 15 مورد تجزیه و تحلیل قرار گرفت. یافته‌ها: نتایج نشانگر افزایش دامنه حرکتی مفصل ران (P=0/05)  در لحظه جدا شدن پنجه پا از زمین در فاز استانس و کاهش درصد استقرار در فاز استانس (P=0/01)  نسبت به قبل از تمرین بود. نتیجه‌گیری: با توجه به نتایج تحقیق، تمرین استقامتی با تقویت عضلات فلکسور و اکستنسور ران موجب افزایش دامنه حرکتی مفصل ران و به تبع آن افزایش درصد استقرار در فاز نوسان راه رفتن افراد سالمند غیر فعال می شود. در نتیجه در نظر گرفتن تمرینات استقامتی در برنامه روزانه افراد سالمند غیر فعال با هدف تقویت تعادل ایستا و افزایش تعادل پویا توصیه می شود.

Title: نقش حمایت‌ های اجتماعی در کاهش اضطراب و افسردگی سالمندان

http://salmandj.uswr.ac.ir/article-1-333-fa.html

اهداف: این مطالعه به منظور نقش انواع حمایت های اجتماعی در کاهش اضطراب و افسردگی سالمندان انجام گرفته است. مواد و روش‌ها: تحقیق حاضر از نوع مطالعات علّی مقایسه ای است که اطلاعات آن با استفاده از پرسشنامه های حمایت اجتماعی نوریک و مقیاس اضطراب و افسردگی در یک نمونه 100 نفری از سالمندان منطقه 2 تهران جمع آوری شد. برای تحلیل داده ها از آزمونهای رگرسیون و ضریب همبستگی استفاده شد. یافته‌ها: یافته های این مطالعه حاکی از آن است که میزان اضطراب و افسردگی در سالمندان به ترتیب 44% و 40% بود. با استفاده از آزمون همبستگی نشان داده شد که تمامی انواع حمایت های اجتماعی (مادی، عاطفی، کارکردی و ساختاری) با اضطراب و افسردگی رابطه معکوس و معنی داری داشتند. نتایج آزمون رگرسیون چند گانه نشان داد که حمایت های مادی و ساختاری پیش بینی کننده های معناداری برای سالمندان دارای اضطراب و حمایت مادی پیش بینی کننده معنادار سالمندان افسرده بود. نتیجه‌گیری: نتایج این مطالعه بر نقش مهم عوامل اجتماعی تعیین کننده سلامت به ویژه حمایت های اجتماعی در اختلالات روانی در سالمندان تاکید دارد. توجه به این منبع ارزان اقتصادی برای مقابله با اضطراب و افسردگی در سالمندان و در نتیجه بهبود کیفیت زندگی آنان از اهمیت شایانی برخوردار می باشد.

Title: شیوع عوامل خطر بیماری‌های قلبی و عروقی در سالمندان امیرشهر در سال 1386

http://salmandj.uswr.ac.ir/article-1-332-fa.html

اهداف: این مطالعه به منظور بررسی شیوع عوامل خطر بیماریهای قلبی و عروقی در سالمندان امیر شهر انجام شد. مواد و روش‌ها: این مطالعه مقطعی بر روی سالمندان بالای 60 سال امیرشهر انجام شد. در امیرشهر حدود 2000 نفر (%5/53 مرد و %5/46 زن) افراد بالای 60 سال زندگی می کنند که حدود 1019 نفر از آنها از طریق نمونه گیری خوشه ای وارد مطالعه شدند. اطلاعات با استفاده از پرسشنامه استاندارد که شامل 22 سوال در دو بخش اطلاعات دموگرافیک و عوامل خطر بیماریهای قلبی و عروقی بود، توسط افراد آموزش دیده و با پرسش از سالمندان و با افراد مطلع خانواده جمع آوری شد. یافته‌ها: آزمودنی ها تعداد 604 نفر (%59/3) مرد و 415 نفر (%40/7) زن بودند. میانگین سن افراد در این بررسی 70±7 سال بود (دامنه 97-60 سال). بیشتر افراد (%55) در گروه سنی 74-65 سال قرار داشتند. در این بررسی فراوان ترین عوامل خطر بیماری‌های قلبی و عروقی به‌ترتیب اضافه وزن و چاقی (%44/1)، فشار خون بالا (%23/9)، دیابت (23/5) و استعمال دخانیات (18%) بود. فشار خون بالا (P=0/07)، دیابت، اضافه وزن و چاقی در بین زنان سالمند (P=0/000) و استعمال دخانیات در بین مردان شایعتر بود (P=0/000). 71/1% افراد سالمند 2 یا 3 عامل خطر ابتلا به بیماری های قلبی عروقی را همزمان داشتند. %25 سالمندان تحت پوشش هیچ بیمه ای نبودند. نتیجه‌گیری: یافته های این مطالعه نشان دهنده شیوع عوامل خطر قلبی و عروقی در افراد مسن بود. پایین بودن فشار خون بالا در این مطالعه نیازمند بررسی دقیق تر و انجام روش های غربالگری جهت کشف بیماران بدون علامت بود.

Title: تاثیر استفاده از ارتز مرتعش بر تعادل ایستا در بیماران مبتلا به پارکینسون

http://salmandj.uswr.ac.ir/article-1-331-fa.html

اهداف: از آنجا که فرض شده اعمال تحریک ارتعاشی توسط یک ارتز مرتعش می تواند تعادل بیماران مبتلا به پارکینسون ایدیوپاتیک را بهبود بخشد، این مطالعه با هدف ارزیابی این فرض انجام شد. مواد و روش‌ها: در این مطالعه مقطعی از روش نمونه گیری غیر احتمالی قضاوتی استفاده شد و تعداد 14 نفر بیمار پارکینسونی (12 مرد و 2 زن) با میانگین سنی (±SD) 59/9±9/1 سال در مطالعه شرکت نمودند. با استفاده از دستگاه تعادل بایودکس، شاخص کلی ثبات در دو وضعیت کلی چشم باز و چشم بسته و هر یک در دو شرایط موتور روشن و موتور خاموش ارتز مرتعش مورد ارزیابی قرار گرفتند. از روش آنالیز واریانس و از روش تی زوجی به ترتیب جهت تعیین وجود اختلاف و مقایسه میانگین های مقادیر شاخص کلی ثبات در چهار وضعیت مورد ارزیابی استفاده شد. یافته‌ها: آزمون آنالیز واریانس وجود اختلاف معنادار بین میانگین مقادیر شاخص کلی ثبات در چهار وضعیت مورد ارزیابی را نشان داد (P=0/000). نتایج آزمون تی زوجی بهبود تعادل را در کلیه وضعیتهایی که حالت چشم باز با حالت چشم بسته مورد مقایسه قرار گرفته بودند (P=0/000) و نیز در وضعیت چشم باز و موتور روشن نسبت به چشم باز و موتور خاموش نشان دادند (P=0/013). در وضعیت چشم بسته، تعادل در حالت موتور روشن از حالت موتور خاموش کمتر بود (P=0/934). نتیجه‌گیری: یک ارتز جدید در توانبخشی بیماران پارکینسونی معرفی شد که می تواند ایده های جدیدی را در طراحی و کاربرد ارتزها معرفی کند. نتایج مطالعه موثر بودن ارتز مرتعش در بهبود تعادل بیماران پارکینسونی را در وضعیت موتور روشن نشان دادند ولی در وضعیت موتور خاموش در مورد موثر بودن آن اطمینان حاصل نشد.

Title: ناتوانی و مرگ و میر ناشی از ترومادر افراد مسن

http://salmandj.uswr.ac.ir/article-1-330-fa.html

اهداف: هدف این مطالعه چند مرکزی، بررسی عوامل پیش بینی کننده ناتوانی و عوامل دخیل در مرگ و میر در افراد سالمند بود. مواد و روش‌ها: این مطالعه توصیفی آینده نگر با استفاده از اطلاعات جمع آوری شده از بیماران ترومایی که در سالهای 77-78 در یکی از مراکز درمانی سینا، شهدا و فیاض بخش بستری شده بودند، انجام گرفت. تمام افراد بالای 65 سال که نیاز به بستری نداشته و یا در بدو مراجعه به بیمارستان فوت کرده بودند از مطالعه حذف شدند. فرم مخصوص مرکز تروما شامل اطلاعات دموگرافیک بیماران، مکانیسم تروما، نوع و شدت آسیب برای تمامی این بیماران تکمیل شد؛ در زمان بستری سایر اطلاعات شامل اقدامات درمانی انجام گرفته و نتایج کلی (ترخیص، فوت و یا سایر موارد) به این فرمها اضافه گردید. در نهایت این اطلاعات مورد آنالیز قرار گرفت. یافته‌ها: تعداد کل بیماران در این مطالعه 296 نفر با میانگین سنی 71/8 سال بود. تصادف با وسایل نقلیه، شایعترین مکانیسم تروما در این بیماران بود. در طی مطالعه، 18 نفر (%6/1) فوت کردند که بیشترین موارد آن در ICU اتفاق افتاد. نتیجه‌گیری: نوع و میزان آسیب بر اساس معیارهای ISS (شدت آسیب) و GCS (مقیاس سطح هوشیاری گلاسکو) مهمترین فاکتورهای پیش گویی کننده فوت بودند. آمار نشان داد که انجام جراحی عمومی با افزایش مرگ و میر ارتباطی نداشت، این در حالی است که جراحی ارتوپدی در کاهش آن موثر بود.

Title: بررسی شیوع پرفشاری خون و دیابت در سالمندان آسایشگاه کهریزک

http://salmandj.uswr.ac.ir/article-1-329-fa.html

اهداف: هدف مطالعه حاضر بررسی شیوع پرفشاری خون و دیابت نوع دو در سالمندان ایرانی ساکن بنیاد خیریه کهریزک بود. مواد و روش ها: در این مطالعه مقطعی 268 زن و مرد بالای 60 سال مورد مطالعه قرار گرفتند. اطلاعات این مطالعه در حین بررسی سلامت سالانه سالمندان جمع آوری گردید. فشارخون بر اساس معیارهای JNC VII و دیابت بر اساس راهنمای انجمن دیابت آمریکا تعریف شد. یافته‌ها: شیوع خام پرفشاری خون در مردان 55% و در زنان 67% بود. میانگین سن و نمایه توده بدن (BMI) در مبتلایان به پرفشاری خون به طور معناداری بیش از افرادی با فشارخون نرمال بود. شیع دیابت در مردان %21 و در زنان %16 بود. میانگین سن، نمایه توده بدن (BMI)، نسبت به دور کمر به باسن (WHR)، کلسترول تام، LDL کلسترول در دیابتی ها به طور معناداری بیش از افراد نرمال بود. نتیجه‌گیری: میزان نمایه توده بدنی با فشار خون رابطه داشت و در این میان زنان در معرض خطر بیشتر پرفشاری خون قرار داشتند. در عوض مردان در معرض خطر بالاتر ابتلا به دیابت و عوامل همراه آن بودند.

Title: نقش تعاملی ویژگی‌ های شخصیتی و فعالیت بدنی بر تحول روانی اجتماعی سالمندان

http://salmandj.uswr.ac.ir/article-1-328-fa.html

اهداف: هدف تحقیق حاضر، تعیین نقش تعاملی فعالیت بدنی و ویژگی های شخصیتی در تحول روانی–اجتماعی سالمندان بود. مواد و روش‌ها: تحقیق حاضر از نوع پس رویدادی (علی–مقایسه ای) بود که در آن 300 سالمند فعال و غیر فعال شهر تهران با دامنه سنی 60-85 سال از بین افرادی که به طور نظم در فعالیت های بدنی شرکت داشتند و افرادی که در این فعالیت ها شرکت نمی کردند به صورت در دسترس، به عنوان نمونه آماری انتخاب شدند. تمامی سالمندان پرسشنامه تحول روانی–اجتماعی هاولی و پرسشنامه ویژگی های شخصیتی آیزنک را تکمیل کردند. داده ها با استفاده از روش های آمار توصیفی (مانند میانگین و انحراف معیار) و آمار استنباطی (مانند آزمون های تحلیل واریانس عاملی و t مستقل) با نرم افزار SPSS نسخه 5/11 در سطح معنی داری P<0/50 تجزیه و تحلیل شدند. یافته‌ها: نتایج نشان داد 70درصد از سالمندان فعال و 68درصد از سالمندان غیر فعال در دامنه سنی 60-70 سال و 50 درصد از سالمندان فعال و 53/3درصد از سالمندان غیر فعال سطح تحصیلات دیپلم و 46/7درصد از سالمندان فعال و 56/7درصد از سالمندان غیر فعال به لحاظ وضعیت اقتصادی در سطح متوسط قرار داشتند. همچنین نتایج نشان داد که سالمندان فعال دارای ویژگی های شخصیتی برونگرایی و ثبات بالاتری هستند و سالمندان فعال برون گرا و با ثبات در ویژگی تحول روانی–اجتماعی برتر از سالمندان غیر فعال بودند. نتیجه‌گیری: فعالیت بدنی تعامل بین ویژگی های شخصیتی با تحول روانی–اجتماعی را تعدیل می کند. بنابراین، احتمالا سبک زندگی فعال در طول عمر، در تعامل با ویژگی های شخصیتی، تحلیل رفتن آمادگی جسمانی، روانی و اجتماعی زود هنگام در دوران سالمندی را به تعویق می اندازد.
